# Host–Guest Complexation of Itraconazole with Cyclodextrins for Bioavailability Enhancement

**DOI:** 10.3390/pharmaceutics16040560

**Published:** 2024-04-19

**Authors:** Lenuţa-Maria Şuta, Amalia Ridichie, Adriana Ledeţi, Claudia Temereancă, Ionuţ Ledeţi, Delia Muntean, Matilda Rădulescu, Renata-Maria Văruţ, Claudia Watz, Florentin Crăineanu, Denisa Ivan, Gabriela Vlase, Lavinia Stelea

**Affiliations:** 1 Advanced Instrumental Screening Center, Faculty of Pharmacy, Victor Babeş University of Medicine and Pharmacy, 2 Eftimie Murgu Square, 300041 Timisoara, Romania; suta.lenuta@umft.ro (L.-M.Ş.); afulias@umft.ro (A.L.); ionut.ledeti@umft.ro (I.L.); circioban.denisa@umft.ro (D.I.); 2 Department II—Pharmaceutical Technology, Faculty of Pharmacy, Victor Babeş University of Medicine and Pharmacy, 2 Eftimie Murgu Square, 300041 Timisoara, Romania; 3 Faculty of Industrial Chemistry and Environmental Engineering, University Politehnica Timisoara, 2 Victoriei Square, 300006 Timisoara, Romania; 4 Faculty of Medicine, Victor Babeş University of Medicine and Pharmacy, 2 Eftimie Murgu Square, 300041 Timisoara, Romania; muntean.delia@umft.ro (D.M.); radulescu.matilda@umft.ro (M.R.); florentin.craineanu@umft.ro (F.C.); stelea.lavinia@umft.ro (L.S.); 5 Faculty of Pharmacy, University of Medicine and Pharmacy Craiova, 2-4 Petru Rares Str., 200349 Craiova, Romania; renata.varut@umfcv.ro; 6 Department I—Pharmaceutical Physics, Faculty of Pharmacy, Victor Babeş University of Medicine and Pharmacy, 2 Eftimie Murgu Square, 300041 Timisoara, Romania; farcas.claudia@umft.ro; 7 Research Centre for Thermal Analysis in Environmental Problems, West University of Timisoara, Pestalozzi Street 16, 300115 Timisoara, Romania; gabriela.vlase@e-uvt.ro

**Keywords:** itraconazole, cyclodextrins, inclusion complex, TG, antifungal activity

## Abstract

Itraconazole is an antifungal agent included in the triazole pharmacological classification that belongs to the BCS class II, characterized by a low solubility in an aqueous medium (of 1 ng/mL, at neutral pH), which is frequently translated in a low oral bioavailability but with a high permeability. In this sense, it is necessary to find solutions to increase/improve the solubility of itraconazole in the aqueous environment. The main purpose of this study is the preparation and analysis of five different guest–host inclusion complexes containing intraconazole. Initially, a blind docking process was carried out to determine the interactions between itraconazole and the selected cyclodextrins. The second step of the study was to find out if the active pharmaceutical ingredient was entrapped in the cavity of the cyclodextrin, by using spectroscopic and thermal techniques. Also, the antifungal activity of the inclusion complexes was studied to examine if the entrapment of itraconazole influences the therapeutic effect. The results showed that the active substance was entrapped in the cavity of the cyclodextrins, with a molar ratio of 1:3 (itraconazole–cyclodextrin), and that the therapeutic effect was not influenced by the entrapment.

## 1. Introduction

**Figure 1:**
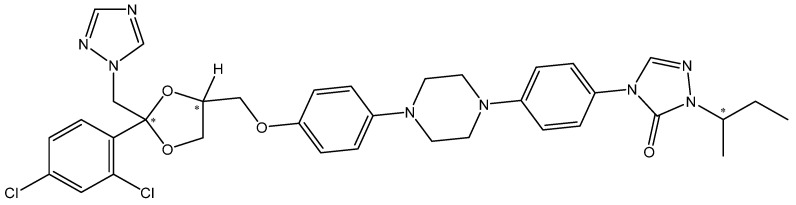
The structural formula of ITZ; * denotes the chiral centers.

Itraconazole (ITZ) is known by its IUPAC name as 2-butan-2-yl-4-[4-[4-[4-[[(2R,4S)-2-(2,4-dichlorophenyl)-2-(1,2,4-triazol-1-ylmethyl)-1,3-dioxolan-4-yl]methoxy]phenyl]piperazin-1-yl]phenyl]-1,2,4-triazol-3-one) and has the molecular formula C_35_H_38_Cl_2_N_8_O_4_ and a molar mass of 705.6 g mol^−1^ [1]. ITZ contains three chiral centers, and it is used in therapy as a mixture of stereoisomers [2] (structure in Figure 1).

The drug presents as a white to slightly yellowish crystalline powder and has a solubility of approximately 1 ng/mL in water, at neutral pH, and as such, it is considered practically insoluble in this solvent [3,4,5]. It is, however, soluble in chloroform and slightly soluble in ethanol and methanol. ITZ has a melting point of 166.2 °C, and it requires a storage temperature of 2–8 °C [6]. It has a pKa of 3.7 and a partition coefficient of 5.66 at a pH value of 8.1, making ITZ a highly lipophilic drug [7]. As a consequence of its acid–base properties, ITZ shows a pH-dependent dissolution profile and requires an acidic gastric environment for appropriate dissolution and absorption [8]. The solubility of ITZ was extensively studied by Vasilev N.A. et al. [9], and the results are presented in comparison to other references from the literature [10,11,12,13], indicating a solubility value of 7.8 × 10^−6^ mol·L^−1^ (i.e., 5.5 µg·mL^−1^) for the drug at pH 1.2 in simulated gastric fluid (SGF). Also, Vasilev et al. [9] reported a theoretical pH-solubility profile of ITZ that was built using the Henderson–Hasselbalch model, suggesting a solubility of 3.4 × 10^−9^ mol·L^−1^ at pH > 5.0, in good agreement with the experimental solubility reported by Bergstrom et al. in 2007, namely 3.3 × 10^−9^ mol·L^−1^ [14]. 

ITZ is an antifungal agent included in the triazole pharmacological classification [15] that belongs to the BCS class II, and it is characterized by a low solubility in an aqueous medium, which is frequently translated in a low oral bioavailability but with a high permeability [16]. The main structural difference between ITZ and other triazoles is the presence of a sec-butyl side chain on the second triazole ring. According to the literature, these structural characteristics can be associated with its biological antifungal and anticancer activities, as well as its interesting pharmacokinetic behavior. ITZ binds strongly to plasma proteins and has a relatively high tissue penetration and a prolonged half-life [17,18]. Itraconazole can be used as a treatment in superficial and systemic fungal infections, given by different species of *Candida* spp. [17,19]. It has also been proven effective against *Cryptococcus* spp. and *Aspergillus* spp., manifesting fungistatic or fungicidal effects depending on the species and strain [20]. 

In this sense, it is necessary to find solutions to increase/improve the solubility of itraconazole in the aqueous environment. Guest–host inclusion complexes formed between hydrophobic molecules and various cyclodextrins are frequently used in pharmaceutical technology since they represent a modern method of increasing the apparent water solubility of the drug, a factor that majorly affects the overall bioavailability of a pharmaceutical formulation [21]. It has been proved that cyclodextrins are able to increase the amount of itraconazole dissolved in intestinal fluids and thus its bioavailability [22,23,24].

Cyclodextrins (CDs) are well known for their solubility-enhancing properties for poorly soluble drugs [18,25,26,27]. Natural CDs are cyclic oligosaccharides containing at least 6 D (+) glucopyranose units attached by a (1–4) glycosidic bond. These cyclic glucopyranose molecules form a truncated cone with a lipophilic inner cavity and a hydrophilic outer surface [28,29,30]. The solubility-enhancing properties of CDs can be better described by a dynamic inclusion mechanism in which lipophilic structures form a complex with the inner cavity of the CDs. In addition, CDs can solubilize compounds by other non-complex related phenomena, for example, by means of drug–cyclodextrin aggregates [4,18,31].

Itraconazole as a cyclodextrin (CD) complex is available in marketed products, and there is a significant volume of data on ITZ-CD. Sporanox^®^, containing itraconazole (ITZ) and 2-hydroxypropyl-β-cyclodextrin (2HPBCD), exemplifies advanced drug delivery by enhancing ITZ’s solubility and bioavailability. This formulation addresses ITZ’s hydrophobicity, facilitating absorption and therapeutic efficacy against a broad spectrum of fungi by inhibiting ergosterol synthesis, showcasing the significance of cyclodextrin complexes in pharmaceutical development [32].

Many researchers assessed the inclusion complex formation between ITZ and β-cyclodextrin (BCD), discovering that BCD notably enhanced ITZ’s solubility in aqueous solutions and supercritical CO_2_. They found higher inclusion yields using the supercritical CO_2_ method over traditional physical mixing and coprecipitation, with temperature and pressure significantly affecting solubility and yield [33]. ITZ was complexed with various CDs to improve its solubility and reduce side effects without compromising antifungal efficacy. The complexes, especially ITR-sulfobutylated β-cyclodextrin sodium salt (SBECD), showed enhanced antifungal activity against Candida albicans. Vaginal bioadhesive tablets developed with ITZ-SBECD proved effective, offering sustained release and improved treatment efficacy [34]. An ITZ solid complex with 2HPBCD and 20% polyvinylpyrrolidone was prepared using co-evaporation, enhancing antifungal efficacy against Candida parapasilosis and Candida albicans. The complex, formulated as a capsule, showed superior dissolution rates to Sporanox^®^, releasing 88% of ITZ in under 30 min. HPLC analysis proved more reliable and precise than antimicrobial and UV-spectrophotometric methods for determining ITZ concentration in the release medium [35]. In another study, the solubilizing effect of 2HPBCD on ITZ was assessed in the presence of bile salts and phosphatidylcholine. Despite these constituents significantly reducing ITZ’s solubility in CDs, ITZ did not precipitate upon adding high concentrations of bile components, contrary to expectations. In dynamic in vitro transfer studies simulating gastrointestinal conditions, ITZ exhibited intestinal supersaturation and concentration-dependent precipitation influenced by bile salts and phospholipids. This highlights how intestinal fluid composition can affect the performance of cyclodextrin-based drug formulations [36]. Other research studies compared hydroxybutenyl-beta-cyclodextrin (HBenBCD) and hydroxypropyl-beta-cyclodextrin (HPBCD) for enhancing itraconazole solubility and bioavailability. HBenBCD significantly increased itraconazole solubility (17-fold) compared to HPBCD (3.8-fold) and showed no food effect in biorelevant media. Oral and intravenous administrations in rats demonstrated that HBenBCD formulations provided higher bioavailability than Sporanox^®^, with solid HBenBCD formulations doubling oral bioavailability compared to Sporanox^®^ solids, without any food effects [37]. In a clinical study, when volunteers ingested ITZ (Sporanox^®^) with 40% 2HPBCD without water, higher duodenal concentrations of the drug were observed, yet systemic exposure remained largely unchanged. This phenomenon was attributed to variations in ITZ entrapment by 2HPBCD in the duodenum. LC-MS/MS analysis confirmed a strong correlation between intestinal concentrations of ITZ and 2HPBCD, indicating that higher 2HPBCD levels lead to less precipitation and more duodenal ITZ. However, this increased complexation reduced the free drug fraction, explaining why higher intraluminal concentrations did not enhance systemic uptake. Thus, understanding 2HPBCD levels in intestinal fluids is vital for interpreting the behavior of cyclodextrin-based oral solutions [38]. Another study aimed to enhance the solubility of ITZ, a poorly soluble, weakly basic drug, by forming binary and ternary inclusion complexes with BCD and polyvinylpyrrolidone (PVP). Binary complexes were prepared in a 1:2 molar ratio of ITZ to BCD using a co-evaporation method, and ternary complexes were formed by adding PVP. Solid-state analyses, including Fourier transform infrared spectroscopy, powder X-ray diffraction, and differential scanning calorimetry, were performed on all formulations and pure ITZ. Solubility tests revealed that both binary and ternary complexes significantly increased ITZ solubility compared to the pure drug, with the ternary complexes showing fast and extensive drug release [39]. Cuoco et al. [40] explored the impact of biomimetic media on ITZ absorption from Sporanox^®^, which contains HPBCD to improve solubility. Findings revealed that bile salts compete with ITZ for HPBCD, affecting solubility and absorption. Higher bile salt concentrations led to quicker supersaturation collapse and reduced permeation, indicating a potential negative food effect, aligning with clinical observations [40].

The main goal of the present study is the preparation and analysis of five different guest–host inclusion complexes containing ITZ as an active pharmaceutical compound. The first goal of the study was to perform molecular modeling to find out which chemical interactions occur between ITZ and the selected CDs. Subsequently, the complexes were analyzed using spectroscopic techniques, namely ATR–FTIR, and thermoanalytical methods (TG/DTG/HF) in order to confirm the entrapment of ITZ in the cavity of the CD. Ultimately, the antifungal activity of the complexes was determined to examine how the entrapment of the active pharmaceutical ingredient influenced the therapeutic effect.

## 2. Materials and Methods

### 2.1. Molecular Modelling

Blind docking process was carried out to determine the interactions between ITZ and β–cyclodextrin (BCD), 2-hydroxypropyl-β-cyclodextrin (2HPBCD), 2-hydroxypropyl-gamma-cyclodextrin (2HPGCD), random methyl-β-cyclodextrin (RAMEB), and sulfobutylated β-cyclodextrin sodium salt (SBECD). 

The virtual 3D structures of the cyclodextrins were obtained in different ways. The structure of BCD (PDB ID: 6JEQ, X-ray diffraction, resolution 1.8 Å) and the GCD (PDB ID: 2ZYK, X-ray diffraction, resolution 2.50 Å) were downloaded from the Protein Data Bank in pdb file format. The cyclodextrins 2HPBCD and 2HPGCD derivatives were drawn manually, using the initial structures of BCD and GCD.

The randomly methylated β-cyclodextrin structure used in this work was generated from the curated coordinates of ligand 6JEQ, and methyl groups were manually added to free hydroxyl groups to obtain a degree of substitution equal to 12 (GaussView 9, Semichem Inc., Shawnee Mission, KS, USA). Substituents were added to the β-cyclodextrin natural core, namely 4 -CH_3_ on the O-2 position for the 2, 3, 4, and 6 glucopyranose units; 5 groups on the O-3 for the 1, 2, 4, 5, and 7 glucose residues; and finally 3 -CH_3_ on 1, 5, and 7 glucopyranose units’ O-6 position. 

For SBECD, the same initial β-cyclodextrin was used, and sodium sulfobutyl groups were manually added to hydroxyl groups from the O-6 position to obtain a degree of substitution equal to 6.

Three-dimensional coordinates of the ITZ guest molecule were generated using the Gaussian program suite. In order to achieve stable, low-energy conformations, ITZ, and CDs were individually optimized at the DFT/B3LYP/6-311G level of theory.

The stoichiometry of the ITZ:CD inclusion complexes was determined in a previous study using the experimental kneading method, from which the possibility of a 1:3 molar ratio of ITZ:CD emerged. As one molecule of ITZ was expected to accommodate three molecules of CDs, consecutive docking cycles were carried out, with each cycle starting from the best result selected from its previous cycle.

The first cycle aimed to define the relative position between ITZ and CD, and the second cycle aimed to define the position of the second and third CDs using a receptor from the first selected result (ITZ/CD 1:1 complex). Finally, in the last docking, we used ITZ as a ligand and CDs as receptors to obtain the best configuration of the complex and to compute the total energy of affinity (kcal/mol). 

The molecular docking analysis was performed using the AutoDock 4.2.6 software together with AutoDockTools 4. The docking between ITZ and CDs involved adding all the polar hydrogens and computing the Gasteiger charge; the grid box was created using Autogrid 4 with 40 × 40 × 40 Å in x, y, and z directions with 0.375 Å spacing from the CD center. For the docking process, we chose the Lamarckian genetic algorithm with a population size of 150 and a number of 30 runs. All other parameters were used with the default values.

The calculations were realized in duplicate, and the results are expressed as averages. For the final visualization stage of the formed inclusion complexes, the PyMol (The PyMOL Molecular Graphics System, Version 2.0 Schrödinger, LLC, New York, NY, USA) software and Discovery Studio Visualization, Version 4.5 (Biovia) software were selected. 

### 2.2. Samples and Preparation

ITZ was acquired from Sigma-Aldrich (Sigma, Steinheim, Germany), batch no. PHR1834, and the cyclodextrins were obtained from CycloLab R&D Ltd. (Budapest, Hungary). All the substances were used as received, without any further purification.

The complexes were prepared using the wet kneading method with absolute ethanol (Honeywell, Riedel-de Haen, Germany) as the solvent and using itraconazole and five cyclodextrins, namely BCD, 2HPBCD, 2HPGCD, RAMEB, and SBECD in a molar ratio of 3:1; After preparation, the complexes were sieved and dried in an oven at 40 °C for 48 h and then transferred in sealed vials.

### 2.3. ATR–FTIR Analysis

To record the UATR–FTIR spectra, a Perkin-Elmer SPECTRUM 100 instrument (Perkin-Elmer Applied Biosystems, Foster City, CA, USA) was used, with the selected domain being 4000–650 cm^−1^, on a diamond/ZnSe ATR device. The spectrum was created after 64 successive scans at a resolution of 2 cm^−1^. The spectral region of 2300–1900 cm^−1^ did not represent any relevance for the interpretation of the spectra, the existing absorption bands being caused by the noise signal of the ATR crystal.

### 2.4. Thermal Analysis

To obtain the thermoanalytical data (TG/DTG/HF), a Perkin-Elmer DIAMOND TG/HF instrument (Perkin-Elmer Applied Biosystems, Foster City, CA, USA) was used. The samples were heated from ambient temperature to 650 °C by using a heating rate of β = 10 °C min^−1^ and a flow rate of 100 mL min^−1^ of synthetic air.

### 2.5. Antifungal Activity

#### 2.5.1. Fungal Strains

This study included a total of 25 clinical isolates from the culture collection of the Microbiology Department of “Victor Babeş” University of Medicine and Pharmacy Timişoara and two quality control strains. 

The clinical isolates represented 10 *Candida albicans*, 14 *Candida parapsilosis*, and 1 *Candida krusei*, while the reference strains were *Candida parapsilosis* ATCC 22019 and *Candida krusei* ATCC 6258 (Microbiologics, Paris, France). 

The *Candida* strains were isolated on Sabouraud with chloramphenicol agar (Sanimed, Giurgiu, Romania). The identification of all isolates was performed according to the morphological characteristics of colonies, and their biochemical tests were carried out using the automated Vitek 2 system (bio-Mérieux, Marcy-l’Étoile, France). At first, sensitivity to ITZ was tested according to the Clinical Laboratory and Standards Institute (CLSI) and European Committee on Antimicrobial Susceptibility Testing (EUCAST) criteria by determining the minimum inhibitory concentration (MIC) using the Sensititre YeastOne (Thermo Scientific, Cheshire, UK). 

#### 2.5.2. The Antifungal Activity

For study compounds, susceptibility testing was performed using the dilution method according to EUCAST and CLSI (formerly NCCLS) recommendations and other studies. 

Using the 24 h culture on Sabouraud agar, the standardized suspension was prepared with sterile distilled water to a concentration of 0.5 Mc Farland (1–5 × 10^6^ colony-forming units/mL) [41,42]. The final inoculum was obtained from the 0.5 McFarland suspension by diluting 1:10 in distilled water, resulting in a suspension of 1–5 × 10^5^ CFU/mL [41].

To dilute the test compounds, we started with a stock concentration of 3.2 mg/mL in DMSO, which was diluted 1:100 in RPMI 1640 with 2% dextrose medium (Thermo Scientific, Roskilde, Denmark) in order to obtain the dilution series of 0.03–16 μg/mL [41]. 

In 10 test tubes, 100 μL from each of the corresponding concentrations of tested compounds and 100 μL suspension of the microorganism under study were added (final inoculum was approximately 0.5–2.5 × 10^5^ CFU/mL, and the final concentration of the compounds was between 0.015 and 8 μg/mL). 

The positive control test contained 100 μL of the RPMI medium, 10 μL DMSO, and 100 μL of the microbial suspension. For negative control, one test tube with 100 μL RPMI medium and 100 μL distilled water was used. 

All study test tubes were incubated at 35 °C in the ambient atmosphere for 24 h.

To determine the minimum inhibitory concentration (MIC) and the minimum fungicidal concentration (MFC), a volume of 1 µL from all test tubes was inoculated with a loop (NuovaAptaca SRL, Canelli, Italy) on Sabouraud with chloramphenicol agar. The MIC of antifungal drugs was considered the lowest concentration resulting in the inhibition of growth of ≥50% of that of the positive control [41], while the MFC was the lowest concentration that killed 99.9% of the microorganisms [41,42,43,44].

#### 2.5.3. Limitations of the Study 

The set of isolates included only Candida species, and all these strains were sensitive to itraconazole.

## 3. Results and Discussion

### 3.1. Molecular Modeling

It is known that the scoring function of AutoDock is derived from the AMBER force field and includes five components for atom–atom interactions as well as an estimate of the conformational entropy loss upon binding (∆*S_conf_*). The atom–atom interactions include the following: dispersion/repulsion term, which is a typical LenardJones 6/12 potential for van der Waals interactions; directional H-bond term based on a 10/12 potential; coulombic potential to estimate electrostatic interactions; and desolvation potential energy estimated based on the volume of atoms that surround a given atom and shelter it from the solvent, weighted by a solvation parameter and exponential term with a distance-weighing factor.

$$∆G=\left(V_{bound}^{L-L}-V_{unbound}^{L-L}\right)+(V_{bound}^{T-T}-V_{unbound}^{T-T})+(\;V_{bound}^{T-L}-V_{unbound}^{T-L}+∆S_{conf})$$

where *T* refers to the protein, *L* refers to the ligand, and *V* is the pair-wise evaluation mentioned above. 

Molecular modeling was used for interaction characterization between ITZ and different CDs in a 1:3 molar ratio.

After AutoDock 4.2.6 redocking, we calculated RMSD, obtaining low RMSD values (all of them being ≤0.3 Å), which suggests that our docking methodology is robust.

The binding free energy values calculated as follows: −10.55 kcal mol^−1^ for the ITZ/BCD inclusion complex, −6.86 kcal mol^−1^ for ITZ/2HPBCD inclusion complex, −7.47 kcal mol^−1^ for ITZ/2HPGCD inclusion complex, −10.74 kcal mol^−1^ for ITZ/RAMEB inclusion complex, and −7.35 kcal mol^−1^ for ITZ/SBECD inclusion complex.

According to our data, the ITZ/RAMEB inclusion complex is the most stable, followed by the ITZ/BCD, with a lower value of binding free energy. 

**Figure 2:**
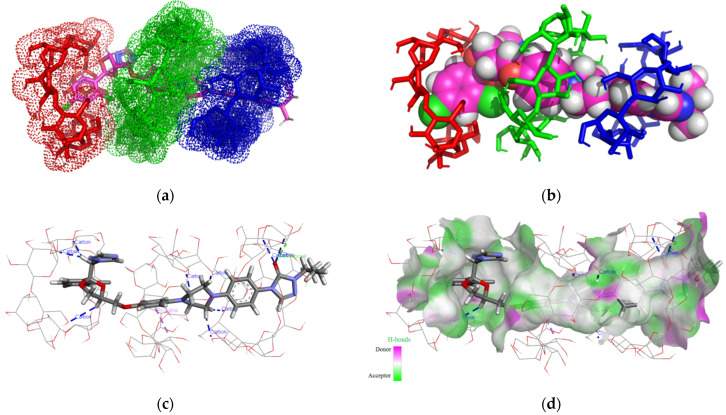
Inclusion complex simulation for a 1:3 molar ratio between ITZ and BCD. Images (**a**,**b**) show the inclusion complex between ITZ and BCD trimer. The ITZ guest molecule is represented in sticks colored by element, while BCDs are represented in red/green/blue dots (**a**); ITZ is represented in spheres colored by element, and BCDs are illustrated as red/green/blue-colored sticks (**b**). Image (**c**) shows polar/hydrophobic contacts between ITZ and BCDs, with ITZ colored by element and BCDs shown in lines. Image (**d**) shows H-bond surface interaction ITZ/BCDs.

Figure 2a–d present the theoretical ITZ/BCD inclusion complexes, as rendered in the PyMOL and Discovery Studio molecular visualization systems, simulated in a 1:3 molar ratio.

Analyzing the 3D images of the ITZ:BCD complex (1:3), we can see that BCDs form a trimer face-to-face assembly macrocycle, tail-to-tail/head-to-tail organization (from left to right), where head means the secondary rim of BCD and tail the primary rim.

ITZ generates nine non-classical hydrogen bonds with the BCD trimer, which involves chlorine, the oxygen from the 5-oxo-1,5-dihydro-4H-1,2,4-triazol radical, and the hydrogen atoms from the ITZ piperazine ring and BCD carbohydrate residues, with the bonds having lengths between 1.65 and 3.53 Å. 

Between ITZ and the middle BCD, a Pi-sigma interaction is established with lengths of 3.54 Å, involving the methoxyphenyl ring and the second carbon from BCD carbohydrate residues. Between ITZ and the right BCD, classical hydrogen bonds appear with lengths of 2.58 Å, involving the carbonyl oxygen of the 1,2,4-triazol-3-one ITZ radical and the hydroxyl hydrogen from the third position of BCD.

**Figure 3:**
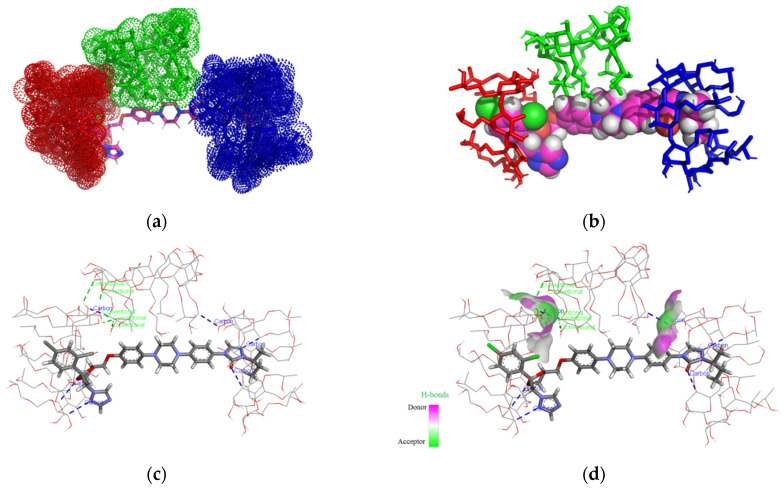
Inclusion complex simulation for a 1:3 molar ratio between ITZ and 2HPBCD. Images (**a**,**b**) show the inclusion complex for the head-to-head-to-tail trimer CD organization of 2HPBCD cavities. The ITZ guest molecule is represented in sticks colored by element, while 2HPBCD molecules are represented in red/green/blue dots (**a**); ITZ is represented in spheres colored by element, and 2HPBCDs are shown in red/green/blue-colored sticks (**b**). Image (**c**) shows polar/hydrophobic contacts between ITZ and 2HPBCD, with ITZ colored by element and 2HPBCDs presented in lines. Image (**d**) shows H-bond surface interaction in ITZ/2HPBCDs.

In the case of the ITZ:2HPBCD complex (1:3) (Figure 3a–d), we can see that the ITZ molecule is included in two 2HPBCD cavities from the secondary face, having four non-classical hydrogen interactions between ITZ 1,2,4-triazol second nitrogen, 1,3-dioxolan’s first oxygen heterocycle, and the carbonyl oxygen of 1,2,4-triazol-3-one with carbon atoms from CD carbohydrate fragments and lengths of 2.85 Å, 3.11 Å, 3.17 Å, and 3.56 Å. The second molecule of 2HPBCD interacts through five classical hydrogen bonds, with lengths between 1.67 and 2.23 Å and one non-classical bond (2.78 Å) with 2HPBCD on the left and one non-classical hydrogen bond with 2HPBCD on the right with lengths of 3.29 Å. The third molecule of 2HPBCD (middle) does not interact with ITZ, as the ligand is not included in the CD cavity.

**Figure 4:**
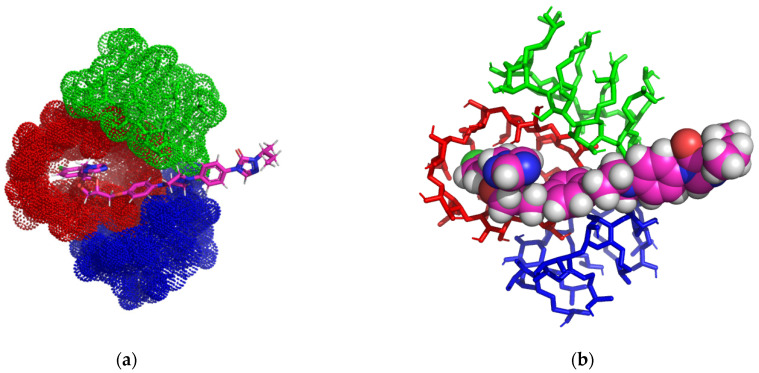

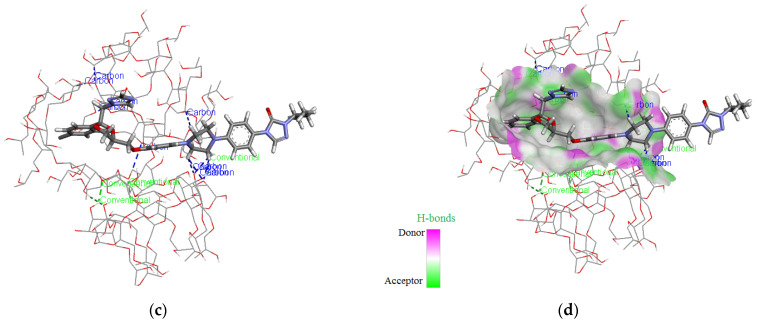
Inclusion complex simulation for a 1:3 molar ratio between ITZ and 2HPGCD. Images (**a**,**b**) show the inclusion complex for the head-to-head-to-head trimer CD organization of 2HPGCD cavities. ITZ guest molecule is represented in sticks colored by element, while 2HPGCDs are represented in red/green/blue dots (**a**); ITZ is represented in spheres colored by element and 2HPGCDs are shown in red/green/blue-colored sticks (**b**). Image (**c**) shows polar/hydrophobic contacts between ITZ and 2HPGCD, with ITZ colored by element and 2HPGCD presented in lines. Image (**d**) shows the H-bond surface interaction in ITZ/2HPGCD.

In the case of the ITZ: 2HPGCD complex (1:3) (Figure 4a–d), we can see that only one 2HPGCD molecule forms an inclusion complex with ITZ (red CD in image a), and ITZ is included in the cavity of the host molecule, resulting in one non-classical hydrogen bond with a length of 2.4 Å between the hydrogen of 1-methyl 1,2,4-triazol radical and hydroxyl oxygen from the third position of a glucopyranoside moiety.

ITZ forms five non-classical hydrogen bonds with the other two CD molecules, with distances of 2.34–3.03 Å, and they do not form inclusion complexes.

The three molecules of 2HPGCD form a trimer stabilized by five classical hydrogen bonds (2.5–2.82 Å) and seven non-classical hydrogen bonds (2.63–3.78 Å). 

**Figure 5:**
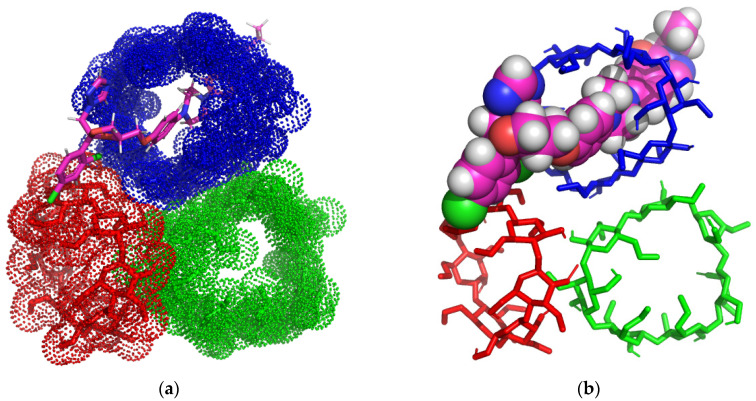

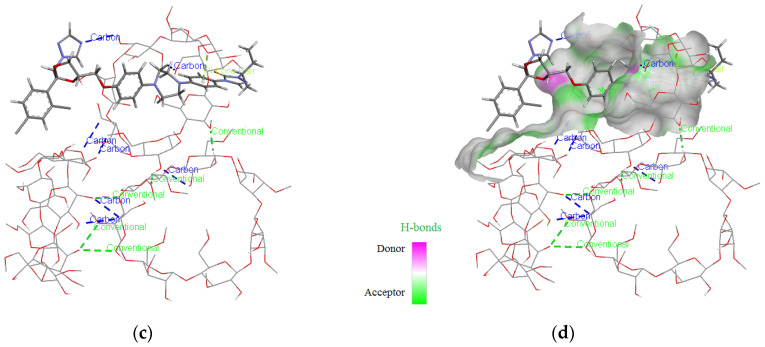
Inclusion complex simulation for a 1:3 molar ratio between ITZ and RAMEB. Images (**a**,**b**) show the inclusion complex for the trimer CD organization of RAMEB cavities. ITZ host molecules are represented in sticks colored by element, while RAMEB molecules are represented in red/green/blue dots (**a**); ITZ is represented in spheres colored by element and RAMEB in red/green/blue-colored sticks (**b**). Image (**c**) shows polar/hydrophobic contacts between ITZ and RAMEB, with ITZ colored by element and RAMEB presented in lines. Image (**d**) shows the H-bond surface interaction in ITZ/RAMEB.

ITZ forms an inclusion complex with one molecule of RAMEB (Figure 5a–d), generating two carbon–hydrogen bonds: the first with a length of 2.67 Å, which involved the hydrogen atoms in position 2 of the piperazine residue and the oxygen of the primary hydroxyl group of a carbohydrate residue, and the second with a length of 3.27 Å, formed between the nitrogen atom in position 3 belonging to the 1,2,4-triazole residue and the primary hydroxyl from a carbohydrate residue. Also, between the ITZ and the same RAMEB molecule, there is a Pi-lone pair interaction with a distance of 2.82 Å, which involves the benzene nucleus and the secondary hydroxyl residue from the third position of a carbohydrate residue.

The three RAMEB molecules are arranged in the same plane, interacting with each other through five classical hydrogen bonds (2.2–3.30 Å) and six carbon–hydrogen bonds (2.83–3.78 Å).

**Figure 6:**
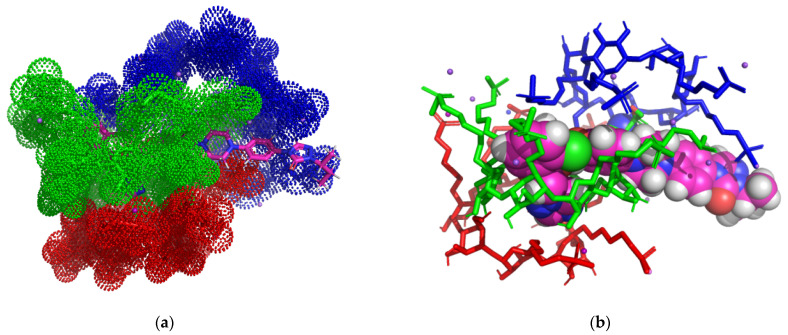

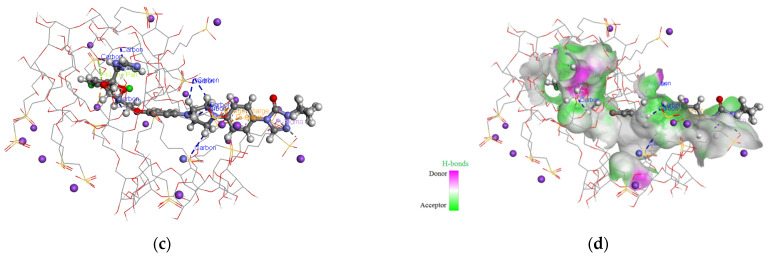
Inclusion complex simulation for a 1:3 molar ratio between ITZ and SBECD. Images (**a**,**b**) show the inclusion complex for the tail-to-tail-to-tail trimer CD organization of SBECD cavities. ITZ host molecules are represented in sticks colored by element, while SBECDs are represented in red/green/blue dots (**a**); ITZ is represented in spheres colored by element and SBECDs in red/green/blue-colored sticks (**b**). Image (**c**) shows polar/hydrophobic contacts between ITZ and SBECD, with ITZ colored by element and SBECDs presented in lines. Image (**d**) shows the H-bond surface interaction in ITZ/SBECD.

Analyzing the 3D images of the ITZ/SBECD interactions in a 1:3 molar ratio (Figure 6a–d), it is observed that ten non-classical hydrogen bonds are generated through host–guest interactions, comprising eight carbon–hydrogen bonds (1.67–2.93 Å) and two Pi-donor interactions (2.90 Å, 4.10 Å).

Between ITZ and SBECD, two Pi-cation electrostatic charge interactions are also observed (2.86 Å and 3.27 Å), involving the benzene nuclei of ITZ and nearby sodium cations, as well as one Pi-sigma hydrophobic interaction (3.98 Å), involving the 1,2,4-triazol-3-one heterocycle and one Pi-lone peer interaction (2.89 Å). SBECD molecules are arranged with the primary faces toward ITZ.

### 3.2. ATR–FTIR Analysis

**Figure 7:**
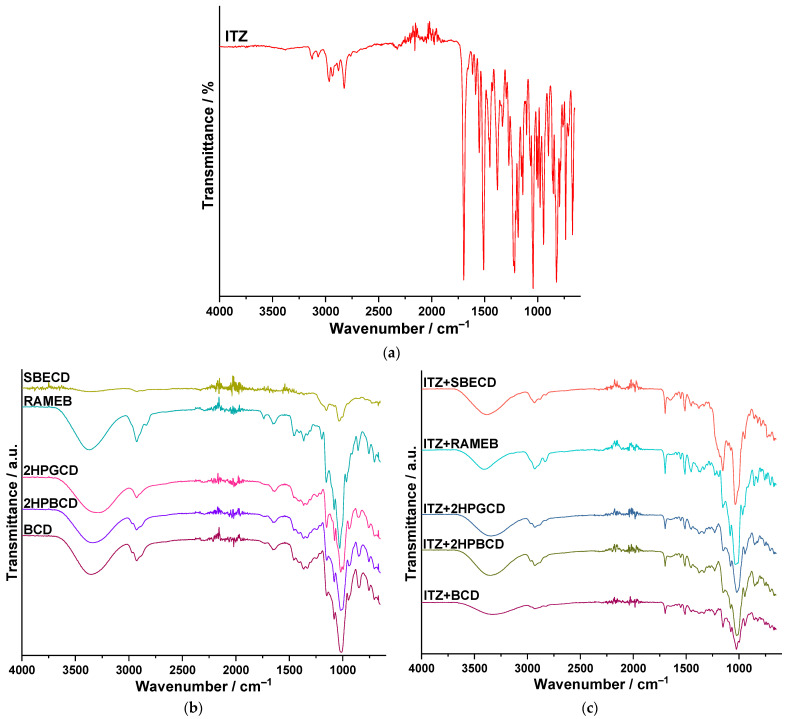
The obtained spectra for (**a**) ITZ, (**b**) CDs, and (**c**) the inclusion complexes.

Figure 7a–c present the spectra obtained for ITZ, CDs, and the inclusion complexes. In order to establish the entrapment of the active pharmaceutical ingredient in the cavity of cyclodextrin, the spectra of the pure substances were compared to the one obtained for the inclusion complex. The method provides information regarding the involvement of hydrogen in various functional groups by determining shifts in the absorption bands to a lower frequency and increases in the intensity, as well as revealing a widening of the band caused by the stretching vibration of the group involved in the formation of the hydrogen bonds [45].

The methyl group from the sec-butyl radical is associated with two absorption bands caused by stretching vibrations: the symmetrical ones at 2879 cm^−1^ and the asymmetrical ones at 2965 cm^−1^. As well, the bending vibrations of the same group are noticed at 1451 cm^−1^ (asymmetrical) and 1379 cm^−1^ (symmetrical). For the methylene groups, the stretching vibrations are observed at 2824 cm^−1^ (symmetrical) and 2934 cm^−1^ (asymmetrical). The scissoring vibration for this group is overlapped by the bending vibration of the methyl radical, and the twisting and wagging vibrations for CH_2_ cannot be observed due to their low intensity. The stretching vibration of the methylene group of the rings (dioxolan and piperazine) reveals an absorption band at 3072 cm^−1^. The stretching bands of the C-H moiety from the benzene nucleus are shown at 3125 cm^−1^. The out-of-plane bending of the C-H bonds of the ring causes a strong and sharp absorption band at 823 cm^−1^. The stretching vibration of the ketone moiety is noticed as an intense absorption band at 1697 cm^−1^. The asymmetrical stretching vibration (C-O-C) of the ether group from the ring is observed at 1140 cm^−1^, while the stretching vibrations of the aryl-alkyl-ether appear at 1217 cm^−1^ (asymmetrical) and 1067 cm^−1^ (symmetrical). The stretching vibration of the C-Cl moiety is observed at 1043 cm^−1^ as a strong absorption band. For the triazole ring, at 1184 cm^−1^, an intense absorption band can be noticed, which is caused by the C-N moiety, and the C=N moiety is also associated with a strong band at 1509 cm^−1^.

For all the CDs, a broad absorption band can be observed in the spectral region of 3700–3025 cm^−1^, determined by the stretching vibrations of the -OH group, which also presents an intermolecular hydrogen bonding; the peaks are as follows: 3376 cm^−1^ (SBECD), 3369 cm^−1^ (RAMEB), 3297 cm^−1^ (2HPGCD), 3336 cm^−1^ (2HPBCD), and 3350 cm^−1^ (BCD). Also, the stretching vibration of the C–O moiety (from alcohols) is observed in all five CDs at approximately 1025 cm^−1^. The stretching vibrations of the methyl and methylene groups are shown in the spectral region of 3000–2780 cm^−1^, and the peaks are as follows:

- SBECD: 2926 and 2874 cm^−1^;
- RAMEB: 2927 and 2838 cm^−1^;
- 2HPGCD: 2928 cm^−1^;
- 2HPBCD: 2973; 2929 and 2887 cm^−1^;
- BCD: 2970; 2927 and 2882 cm^−1^.

The bending vibrations of the methyl group are also highlighted as follows:

- SBECD: 1368 cm^−1^ (symmetrical) and 1458 cm^−1^ (asymmetrical);
- RAMEB: 1365 cm^−1^ (symmetrical) and 1459 cm^−1^ (asymmetrical);
- 2HPGCD: 1365 cm^−1^ (symmetrical) and 1459 cm^−1^ (asymmetrical);
- 2HPBCD: 1365 cm^−1^ (symmetrical) and 1459 cm^−1^ (asymmetrical);
- BCD: 1365 cm^−1^ (symmetrical) and 1457 cm^−1^ (asymmetrical).

In the case of the inclusion complex formed between ITZ and SBECD, the spectra show a broadening of the bases of most of the absorption bands, especially in the region of 3675–3020 cm^−1^, due to the hydrogen bonds formed between the CD and the active pharmaceutical ingredient. In addition, the absence of low-intensity absorption bands of ITZ is also observed, along with the remarkable reduction in the intensity of the strong ones (1698; 1510; 823 cm^−1^). The absorption band of SBECD (2926 cm^−1^) and that of ITZ at 2934 cm^−1^ merge, creating a new peak at 2932 cm^−1^. These aspects suggest that ITZ is entrapped in the cavity of the CD, namely SBECD. 

Regarding the complex of ITZ+RAMEB, the broadening of some absorption bands is observed, especially in the spectral region of 3033–2765 cm^−1^ and the band described by the value of ν = 1035 cm^−1^. Also, multiple peaks of the CDs are shifted, for example, 3368 → 3408 cm^−1^, 2838 → 2832 cm^−1^, 1459 → 1452 cm^−1^, and 1029 → 1035 cm^−1^. Most of the absorption bands in ITZ are either not noticeable, or they present a significantly reduced intensity (1699, 1551, and 1511 cm^−1^). Given the results of the ATRTIR analysis, the formation of the inclusion complex between the two compounds is confirmed (ITZ+RAMEB).

For the complex formed between ITZ and 2HPGCD, it can also be noted that some absorption bands present a wider base, there is a reduction in the intensity of the intense bands of ITZ (1698, 1510, and 825 cm^−1^), and some peaks characteristic of 2HPGCD are shifted. The lack of the majority of the absorption bands of the active substance indicates its entrapment in the CD cavity. 

Concerning the inclusion complex between ITZ and 2HPBCD, the widening of some peak bases can be observed, especially in the fingerprint region. The shifting of some bands can also be noticed for 2HPBCD: 3336 → 3347 cm^−1^, 2887 → 2879 cm^−1^, and 1241 → 1230 cm^−1^. For ITZ, the significantly reduced intensity of the peak is observed at ν = 1698, 1511, and 1451 cm^−1^. Because of these aspects, it can be stated that the inclusion complex between ITZ and 2HPBCD is formed.

As regards the complex of ITZ with BCD, several differences are highlighted. Concerning the CD, besides the shifting of some absorption bands (3350 → 3327 cm^−1^ and 2970 → 2962 cm^−1^), it can be observed that the intense peak at 1012 cm^−1^ of BCD is split on the spectra of the inclusion complex in a boarder absorption band, with two maxima at ν = 1025 cm^−1^ and ν = 999 cm^−1^. The first maximum is represented by a shift from 1012 to 1025 cm^−1^, while the second one is due to a slight increase in the intensity (~8%) of the absorption band at ν = 997 cm^−1^ on the spectrum of ITZ, which may be caused by the hydrogen bonds formed between ITZ and the CD. The reduction in the intensity of the absorption bands for ITZ can be noticed at ν = 1698 cm^−1^, ν = 1510 cm^−1^, and ν = 1378 cm^−1^. These aspects suggest that ITZ is entrapped in the cavity of the CD, namely BCD.

### 3.3. Thermal Analysis

**Figure 8:**
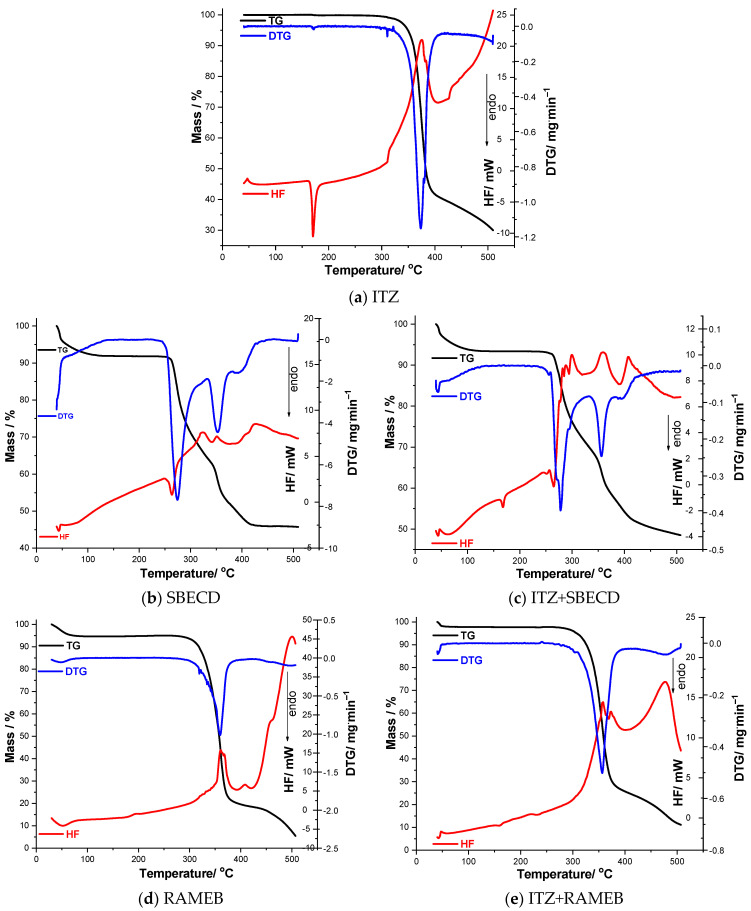

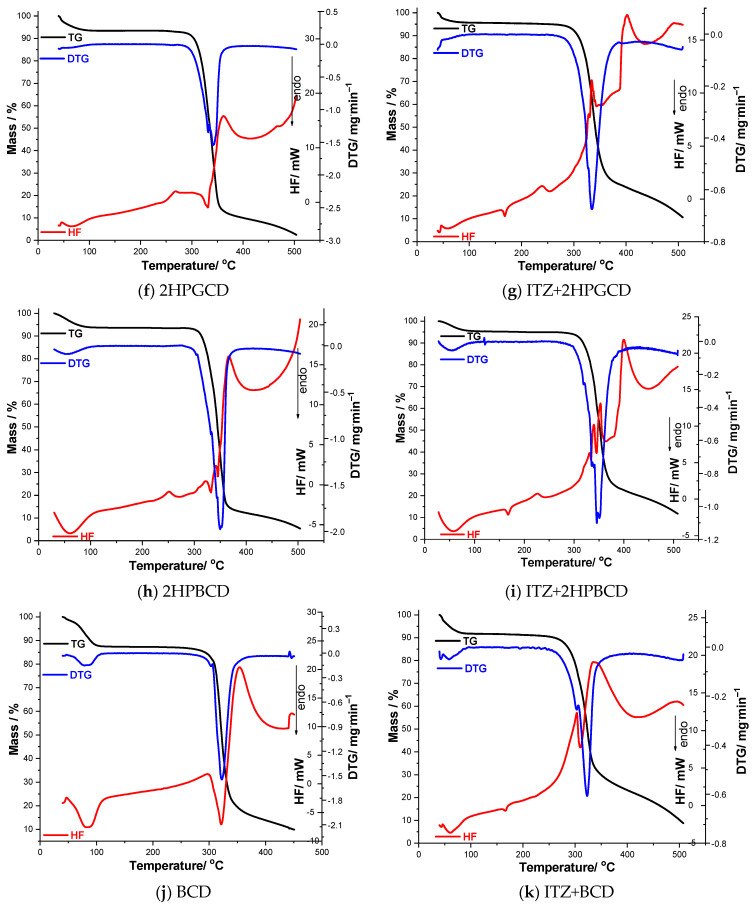
Thermogravimetric curves (TG/DTG/HF) obtained under non-isothermal conditions.

Figure 8a–k and Table 1 show the results of the thermal analysis (TG/DTG/HF) obtained under non-isothermal conditions (using a heating rate of β = 10 °C min^−1^). 

The thermal decomposition of ITZ begins at 305 °C, showing good thermal stability; this is the main degradation process that the active substance undergoes, with a loss of more than half of its mass. This process is also observed on the DTG curve, with a peak at 373 °C. On the HF curve, the first process noticed is an endothermic one, representing the melting point of the active pharmaceutical ingredient, 170 °C; this value is in good agreement with the data presented in the literature [6,7]. The second process on the HF curve is an exothermic one, which starts at 309 °C and presents a peak at 375 °C. 

The decomposition processes of the CDs show many similarities. The first process highlighted by the TG and DTG curves represents dehydration, which is also observed as an endothermic peak on the HF curves of BCD, 2HPBCD, 2HPGCD, and RAMEB. After dehydration, the degradation process of the CDs begins, which occurs at a high temperature, suggesting a high thermal stability of the CDs. The decomposition process of BCD starts at 279 °C (DTG_peak_ = 322 °C), with a loss of mass of 74.8%, which is associated with two exothermic peaks on the HF curve (267 °C and 354 °C). Both 2HPBCD and 2HPGCD show a thermal stability up to ~290 °C and a loss of mass of approximately 85%, each process being associated with two exothermic peaks. The loss of mass of RAMEB starts at 296 °C and ends at 498 °C (DTG_peak_ = 359 °C), with the total Δm being 85.9%. For SBECD, the thermoanalytical results indicate the initial degradation of the CD at a temperature of 244 °C (DTG_peak_ = 274 °C), which ends at 318 °C, Δm = 24.55%. This temperature range on the HF curve is evidenced by an endothermic peak (263 °C) associated with the melting point of SBECD. The second degradation process of the CD starts at 318 °C, which is associated with three exothermic events on the HF curve at 323 °C, 351 °C, and 422 °C. 

In the case of the binary mixtures, the first difference observed on all the HF curves is the diminished intensity of the melting point of ITZ. By studying the obtained TG and DTG curves, it can be observed that, for the inclusion complexes formed between ITZ+BCD, ITZ+2HPBCD, ITZ+2HPBGD, and ITZ+RAMEB, there is a reduction in the temperature at which ITZ begins the degradation process, with the temperature being slightly higher in the case of the binary mixture of ITZ+SBECD. The endothermic processes observed on the HF curve at a temperature of approximately ~50 °C are associated with the dehydration of the CD from the binary mixture. In the case of the inclusion complex ITZ+BCD, it can be observed that HF_peak_ = 336 °C, associated with the degradation of the CD, is shifted to a lower temperature than the one observed for the pure CD. The thermal analysis of ITZ+2HPBCD revealed the presence of new exothermic events on the HF curve. The same results were obtained for the binary mixture of ITZ+2HPGCD. Regarding the inclusion complex of ITZ+RAMEB, on the HF curve, the exothermic event is observed at 477 °C, which shows an increase in the temperature of the same process observed for the pure CD (at HF_peak_ = 408 °C). The major difference noticed for the complex of ITZ+SBECD is the appearance of three exothermic peaks, which are the result of the splitting of the exothermic process observed for the pure CD at HF_peak_ = 323 °C.

Given these results, it can be concluded that the active pharmaceutical ingredient (ITZ) is entrapped in the cavity of the selected CDs (SBECD, RAMEB, 2HPGCD, 2HPBCD, and BCD). 

### 3.4. Antifungal Activity

In Table 2, the results of the MIC and MFC values for the tested strains are presented. 

MIC table color code is as follows:

- The MIC in blue is unchanged compared to the MIC from unassociated ITZ;
- MIC with green is higher but remains sensitive;
- The MIC in yellow is higher than the MIC from unassociated ITZ and is interpreted as dose-dependent sensitivity;
- MIC with pink indicates a lower MIC (better antifungal activity).

The interpretation of the results is according to CLSI and NCCLS recommendations [41,42]:

- Sensitive: MIC ≤ 0.125;
- Sensitivity dependent on dose (low): 0.25 ≤ MIC ≤ 0.5;
- Resistant: MIC ≥ 1.

Comparing the antifungal activity of ITZ with the antifungal activity of the five inclusion complexes, it can be concluded that:

- The inclusion complex of ITZ+SBECD exhibited an increased value of the MIC, without generally influencing the antifungal activity; most of the tested strains were sensitive to this association, and only four strains exhibited dose-dependent sensitivity.
- The inclusion complex of ITZ+2HPBCD showed an increase in the MIC value, without influencing the antifungal activity; most of the tested strains were sensitive to this complex, and only three strains presented dose-dependent sensitivity.
- For the complex of ITZ+RAMEB, although the MIC increased, it did not influence the antifungal activity, with only one strain exhibiting a dose-dependent sensitivity and the other ones remaining sensitive.
- In the case of the inclusion complex of ITZ+2HPGCD, the MIC value remained mostly unchanged, and in one case (CP2 strain), even a lower value for MIC was observed.
- Regarding the inclusion complex of ITZ+BCD, the results predominantly show a constant value of the MIC, with one exception for the CP2 strain, where a lower MIC value was observed.

## 4. Conclusions

In the present study, five inclusion complexes, formed between ITZ and BCD, 2HPBCD, 2HPGCD, RAMEB, and SBECD, were fully characterized with theoretical and experimental methods. The results showed that the stoichiometry of the inclusion complexes was 1:3 (itraconazole–cyclodextrin) and that the active pharmaceutical ingredient was successfully entrapped in the cavity of cyclodextrin after applying the wet kneading method in order to obtain the binary mixture. The instrumental techniques used were ATR–FTIR spectroscopy and thermal analysis (TG/DTG/HF). The antifungal effect was generally maintained after the entrapment; in two cases, an increase was observed in the MIC, namely for the CP2 strain, and a lower MIC value was found for ITZ+2HPGCD and ITZ+BCD. 

## Figures and Tables

**Table 1:** The results of the thermal analysis (TG/DTG/HF) obtained under non-isothermal conditions.

Sample	TG		Δm/%	DTG		HF	
	T_onset_/°C	T_offset_/°C		T_onset_/°C	T_peak_/°C	T_onset_/°C	T_peak_/°C
ITZ	305	403	58.09	314	373	160	170
	403	505	10.53	–	–	309	375
SBECD	39	126	8.04	–	–	–	–
	244	318	24.55	243	274	249	263
	318	415	20.67	333	352	293; 341; 388	323; 351; 422
ITZ+SBECD	40	125	6.52	40	44	40	43
	240	320	21.25	242	278	160; 244; 275	168; 264; 281; 288; 299
	320	390	16.17	330	356	335	359
	390	505	7.29	–	–	391	408
RAMEB	34	78	4.7	34	48	39	51
	296	498	85.9		359	310; 395	362; 408
ITZ+RAMEB	40	70	2.17	40	41	40; 152	42; 160
	270	402	72.08	274	356	317; 367	358; 373
	402	505	17.27	–	–	406	477
2HPGCD	44	85	4.9	–	–	47	67
	288	455	85.4	288	332; 342	237; 331	267; 362
ITZ+2HPGCD	40	90	4.20	46	54	40	58
	90	280	1.31	–	–	160; 213	168; 239
	280	440	74.47	254	334	304; 387	328; 334; 402
2HPBCD	35	86	5.3	39	55; 352	35	59
	298	485	85.8			221; 297	251; 367
ITZ+2HPBCD	30	100	4.56	30	54	30	57
	100	280	0.70	–	–	160; 203	168; 226
	280	450	76.3	277	317; 335; 345; 350	289; 381	330; 340; 354; 400
BCD	42	104	12.1	60	83	48	85
	279	434	74.8	260	322	220; 321	297; 354
ITZ+BCD	40	88	8.06	47	58	40; 156	60; 167
	250	415	68.74	254	302; 323	225; 309	311; 419

**Table 2:** The obtained values of MIC and MFC for the tested strains.

Strains	Antifungal Agent												
	ITZ+RAMEB		ITZ+2HPGCD		ITZ+SBECD		ITZ+2HPBCD		ITZ+BCD		ITZ		
											Study Test		Sensititre
	MIC μg/mL	MFC μg/mL	MIC μg/mL	MFC μg/mL	MIC μg/mL	MFC μg/mL	MIC μg/mL	MFC μg/mL	MIC μg/mL	MFC μg/mL	MIC μg/mL	MFC μg/mL	MIC μg/mL
CP ATCC 22019	0.12	0.25	0.06	0.12	0.12	0.25	0.12	0.25	0.06	0.12	0.06	0.12	0.06
CK ATCC 6258	0.12	0.25	0.06	0.12	0.12	0.25	0.12	0.25	0.06	0.25	0.06	0.12	0.06
CA1	0.06	0.25	0.06	0.12	0.12	0.25	0.12	0.25	0.06	0.12	0.03	0.12	0.03
CA2	0.12	0.5	0.12	0.25	0.12	0.25	0.25	0.5	0.12	0.25	0.12	0.25	0.12
CA3	0.12	0.5	0.06	0.25	0.12	0.5	0.12	0.5	0.06	0.12	0.06	0.12	0.06
CA4	0.12	0.5	0.06	0.12	0.12	0.5	0.12	0.5	0.06	0.25	0.06	0.12	0.06
CA5	0.12	0.5	0.06	0.25	0.12	0.5	0.12	0.5	0.06	0.25	0.06	0.25	0.06
CA6	0.12	0.25	0.06	0.12	0.12	0.25	0.12	0.5	0.06	0.25	0.06	0.12	0.06
CA7	0.12	0.25	0.12	0.25	0.25	0.5	0.25	0.5	0.12	0.25	0.12	0.25	0.06
CA8	0.12	0.25	0.06	0.25	0.12	0.25	0.12	0.5	0.06	0.25	0.06	0.25	0.06
CA9	0.12	0.25	0.03	0.12	0.12	0.25	0.12	0.25	0.03	0.12	0.03	0.12	0.03
CA10	0.12	0.5	0.06	0.12	0.12	0.25	0.12	0.25	0.03	0.12	0.03	0.06	0.03
CP1	0.12	0.25	0.06	0.12	0.12	0.25	0.12	0.25	0.03	0.06	0.03	0.06	0.03
CP2	0.25	0.5	0.06	0.12	0.25	0.5	0.12	0.5	0.06	0.12	0.12	0.25	0.12
CP3	0.12	0.5	0.06	0.25	0.12	0.5	0.12	0.5	0.06	0.12	0.06	0.12	0.06
CP4	0.12	0.5	0.06	0.12	0.12	0.5	0.12	0.5	0.06	0.12	0.06	0.25	0.06
CP5	0.12	0.25	0.06	0.12	0.12	0.5	0.12	0.5	0.06	0.12	0.06	0.12	0.06
CP6	0.12	0.25	0.06	0.12	0.12	0.5	0.12	0.5	0.06	0.12	0.06	0.12	0.06
CP7	0.12	0.25	0.06	0.12	0.12	0.5	0.12	0.5	0.06	0.12	0.06	0.12	0.06
CP8	0.12	0.25	0.12	0.25	0.25	0.5	0.12	0.5	0.12	0.5	0.12	0.25	0.12
CP9	0.12	0.25	0.06	0.12	0.12	0.5	0.12	0.5	0.06	0.25	0.06	0.12	0.03
CP10	0.12	0.25	0.12	0.25	0.12	0.5	0.12	0.5	0.12	0.25	0.12	0.5	0.12
CP11	0.12	0.25	0.06	0.12	0.12	0.5	0.12	0.5	0.06	0.25	0.06	0.12	0.03
CP12	0.12	0.25	0.06	0.12	0.12	0.5	0.12	0.5	0.06	0.12	0.06	0.12	0.06
CP13	0.12	0.25	0.06	0.12	0.12	0.5	0.12	0.5	0.06	0.25	0.06	0.12	0.06
CP14	0.12	0.25	0.12	0.25	0.25	0.5	0.25	0.5	0.12	0.25	0.12	0.25	0.12
CK	0.12	0.25	0.06	0.25	0.12	0.5	0.12	0.5	0.06	0.12	0.06	0.12	0.03

CP—*Candida parapsilosis*, CK—*Candida krusei*, CA—*Candida albicans*.

## Data Availability

Raw data are available upon request from the corresponding author of this work.
